# The Effects of CytoSorb in Critically Ill Adult Patients on Vasopressor Support: A Systematic Review and Meta-Analysis

**DOI:** 10.3390/life16040576

**Published:** 2026-04-01

**Authors:** Martina Giacco, Benedetta Savasta, Andrea Montisci, Federico Pappalardo, Luigi La Via

**Affiliations:** 1 School of Medicine and Surgery, University of Enna “KORE”, 94100 Enna, Italy; martina.giacco@unikorestudent.it (M.G.); benedetta.savasta@unikorestudent.it (B.S.); 2 Division of Cardiothoracic Intensive Care, ASST Spedali Civili, 25123 Brescia, Italy; montisci.andrea@yahoo.it; 3 Department of Medicine and Surgery, University of Enna “KORE”, 94100 Enna, Italy; federico.pappalardo@unikore.it; 4 Centro Cuore GB Morgagni, 95125 Catania, Italy; 5 Department of General Surgery and Medical-Surgical Specialties, University of Catania, 95123 Catania, Italy; 6 Department of Anesthesia and Intensive Care, University Hospital Policlinico “G. Rodolico–San Marco”, 95123 Catania, Italy

**Keywords:** vasoplegia, extracorporeal therapy, shock, cytokine removal, inflammation

## Abstract

Background: The impact of CytoSorb hemoadsorption on hemodynamic stability, mortality, and intensive care unit length of stay in critically ill adult patients requiring vasopressor support is unclear. Methods: Systematic review and meta-analysis of randomized controlled trials and observational studies, enrolling adult intensive care unit patients requiring vasopressor support. We compared CytoSorb hemoadsorption versus standard care or control interventions. Results: Twelve studies enrolling 568 patients met the inclusion criteria. Primary outcomes included noradrenaline dosage, mortality at longest follow-up, and intensive care unit length of stay. CytoSorb treatment significantly reduced noradrenaline requirements (mean difference −0.08 μg/kg/min [95% CI: −0.15 to −0.02], *p* = 0.02, I^2^ = 8%). Mortality at the longest follow-up was lower with CytoSorb (risk ratio 0.66 [95% CI: 0.55–0.80], *p* < 0.001, I^2^ = 0%), though this finding was driven primarily by observational studies; randomized controlled trials alone showed non-significant mortality reduction (risk ratio 0.23 [95% CI: 0.05–1.06], *p* = 0.06). No difference in intensive care unit length of stay was observed (mean difference 0.24 days [95% CI: −1.23 to 1.70], *p* = 0.75). Trial sequential analysis indicated insufficient information size for definitive conclusions. Overall evidence quality was low to very low. Conclusions: CytoSorb hemoadsorption may reduce vasopressor requirements in critically ill patients. Observed mortality benefits were driven primarily by observational studies, with RCTs showing non-significant trends. Overall evidence quality is low, and findings should be considered hypothesis-generating; adequately powered RCTs are needed before clinical recommendations can be made.

## 1. Introduction

The management of critically ill patients with hemodynamic instability remains one of the most challenging aspects of intensive care medicine. Cytokine storm and dysregulated host immune responses are recognized as central pathophysiological mechanisms in various critical conditions including septic shock, cardiogenic shock, and post-cardiopulmonary bypass inflammatory states [1,2]. This exaggerated inflammatory response often manifests as vasoplegic shock requiring high-dose vasopressor support, which is independently associated with adverse outcomes [3,4]. Extracorporeal blood purification therapies have emerged as potential adjunctive interventions to modulate these dysregulated immune responses [5]. Among these, hemadsorption with the Cytosorb adsorber (CytoSorbents Corporation, Princeton, NJ, USA) has gained particular attention due to its capacity to remove middle molecular weight molecules (approximately 5–60 kDa), including various pro-inflammatory and anti-inflammatory cytokines, damage-associated molecular patterns, and pathogen-associated molecular patterns [6,7]. This broad-spectrum adsorption profile theoretically positions Cytosorb as a promising intervention for attenuating the “cytokine storm” observed in various critical illnesses [8]. Moreover, persistent hypotension is a frequent accompanying phenomenon of shock states, requiring high dose of vasoactive and inotropic medication, but their use carries significant risks, including arrhythmias, myocardial ischemia, and digital and splanchnic hypoperfusion [9,10]. Strategies that reduce vasopressor requirements while maintaining hemodynamic targets may therefore confer clinical benefits beyond survival [11]. The vasoactive-inotropic score (VIS) has emerged as a validated tool for quantifying vasoactive support and has been associated with outcomes in various critical care populations [12,13]. In this light, we aimed at evaluating the impact of Cytosorb hemadsorption on hemodynamic stability in adult ICU patients requiring vasopressor support.

## 2. Materials and Methods

The protocol of our systematic review and meta-analysis was registered by two authors on PROSPERO (identified record number CRD420251056712). We followed the PRISMA statement for reporting systematic reviews and meta-analyses and a PRISMA checklist is provided separately (Supplementary Material). The core search was structured by combining the findings from three groups of terms. The first group included the terms “Intensive care unit” OR ICU OR “critical care” OR “intensive care” OR critical OR unstable. The second group contained “hemodynamically unstable” OR hemodynamic* OR mortality OR stability OR “length of stay” OR vasopressor; the third group contained the term “cytosorb”. The search was performed on PubMed and Scopus up to 31 May 2025 to identify the relevant articles. An update of the search was performed on the 1 October 2025. Two further searches were performed manually and independently by two authors, also exploring the list of references of the findings of the systematic search. Language restrictions were applied—only articles written in English were included. Inclusion criteria were pre-specified according to the PICOS approach (Supplementary Material). We excluded case reports, experimental animal studies, book chapters, reviews, editorials and letters to the editor. Studies involving pediatric populations, those comparing Cytorsorb with other cartridges or patients treated outside ICU were also excluded. Study selection for determining the eligibility for inclusion in the systematic review and data extraction were performed independently by two reviewers. Discordances were resolved involving a senior author. Data were inserted in a password protected database on Excel. When essential data were missing or unclear in the selected studies, we contacted the corresponding authors via email with a request for additional information and clarification, allowing two weeks for response before making a final determination on inclusion. We performed a subgroup analysis according to the study design, in order to separate data coming from RCTs from data coming from study with different design. Two types of sensitivity analyses were planned “a priori”: (1) using a “leave-one-out at time” approach (performing several analyses excluding one study at time); (2) excluding studies at high risk of bias.

### Quality Assessment, Publication Bias and GRADE of Evidence

Risk of bias assessment for RCTs was performed by two authors using the Cochrane Rob-2 tool which incorporated the following domains: selection, performance, detection, attrition, performance and other potential sources of bias. We used the Newcastle–Ottawa scale (NOS) for observational studies. According to their score, studies were classified as high-risk (1–3 points), intermediate-risk (4–5 points) and low-risk of bias (6–9 points). Presence of publication bias was investigated by visual inspection of funnel plots and Egger’s test for the primary outcome. Certainty of the evidence was assessed according to the recommendations of the Grading of Recommendations Assessment, Development and Evaluation working group by three authors using the GRADEpro software (version 3.0). Meta-analysis was performed using Review Manager software version 5.4.1 (Revman^®^, The Cochrane Collaboration, London, UK). For a dichotomous outcome we analyzed the number of events over the population sample for both groups. We used the mean and standard deviation (SD) for continuous outcomes results, if reported. In the case of data provided as median and interquartile range, we used the approaches suggested by Luo et al. and Wan et al. A random effect model was used, the differences for the continuous outcomes and the categorical variables were analyzed using the inverse variance (IV) method with a 95% or confidence interval (CI). Results were reported in terms of mean difference (MD) or risk ratio (RR), and *p* values were two-tailed and considered significant if <0.05. The presence of statistical heterogeneity was assessed using the X2 (Cochran Q) test. Heterogeneity was likely if Q > df (degrees of freedom) suggested and confirmed if *p* ≤ 0.10. Quantification of heterogeneity was performed and values of I2 ranging 0–24.9%, 25–49.9%, 50–74.9% and >75% were considered as none, low, moderate and high heterogeneity, respectively. Two authors performed trial sequential analyses (TSAs) on the investigated outcomes using the freely available TSA Software 0.9.5.10 Beta version (Copenhagen Trial Unit’s TSA Software^®^; Copenhagen, Denmark). The information size was computed assuming an alpha risk of 5%, a beta risk of 20%. The estimated effects were computed averaging results of the classical meta-analysis method. Further details on interpretation of TSA are available elsewhere [14,15].

## 3. Results

**Figure 1:**
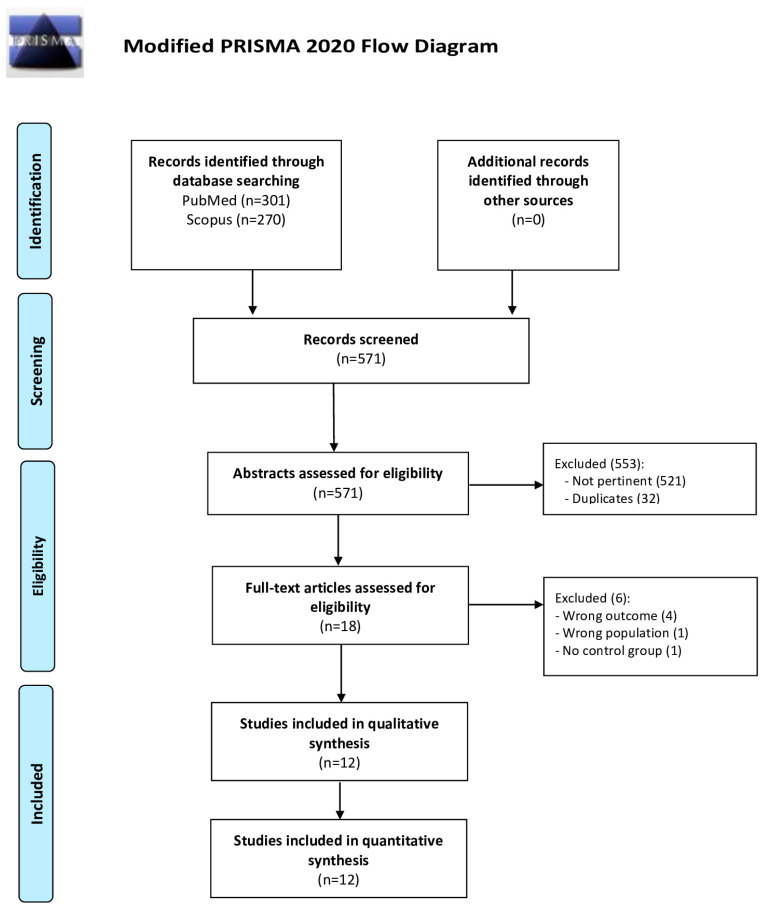
PRISMA flow diagram [28].

Our search identified 301 findings via PubMed and 270 via Scopus, therefore a total of 571 records were screened, as shown in the PRISMA flow diagram (Figure 1).

After the initial title and abstract selection, 521 studies were excluded as they were not pertinent to the focus of the systematic review and 32 were removed as duplicates. Therefore, we assessed the full text articles of the 18 remaining studies according to the PICOS criteria, thus finally including 12 articles for qualitative and quantitative synthesis. The characteristics of the included studies are provided in Table 1.

Four randomized controlled trials [16,17,23,24] were identified, all of which were single-center studies. The remaining eight studies comprised observational designs including retrospective matched cohorts and prospective observational studies. One case series without a control group was excluded [27]. The clinical settings varied considerably, with seven studies conducted in cardiac surgery patients (predominantly during cardiopulmonary bypass), while others examined septic shock (n = 3), cardiogenic shock with VA-ECMO (n = 1), and severe acute pancreatitis (n = 1). Primary outcomes varied across studies, though hemodynamic parameters (particularly vasopressor requirements or vasoactive-inotropic scores) were the most common endpoints. Only one study [7] designated mortality as the primary outcome. Among the investigated outcomes, we were able to analyze only hemodynamic stability, mortality and ICU LOS, as the other planned outcomes (plasmatic cytokine reduction, PCR reduction, PCT reduction) were only reported in less than three studies. Four studies reported data on hemodynamic stability after Cytosorb treatment. Stability was defined as a reduction in noradrenaline dosage after Cytosorb, as compared to the control group providing no treatment. The hemoadsorption treatment duration and norepinephrine assessment timepoints varied across studies. Haidari et al. [19] applied CytoSorb therapy for a median duration of 24 h (range 10–48 h) with norepinephrine requirements assessed at baseline, 12, 24, and 48 h after treatment initiation. Hawchar et al. [17] implemented a standardized 24 h treatment protocol with vasopressor dose measurements at baseline, 6, 12, and 24 h. Mehta et al. [26] utilized CytoSorb for 24 h with vasopressor assessments performed at 0, 6, 12, and 24 h after intervention. Nemeth et al. [16] administered hemoadsorption throughout the intraoperative period with extended postoperative treatment if clinically indicated (maximum 24 h total), measuring norepinephrine requirements at baseline, end of surgery, and 24 h postoperatively.

### 3.1. Noradrenaline Dosage

**Figure 2:**
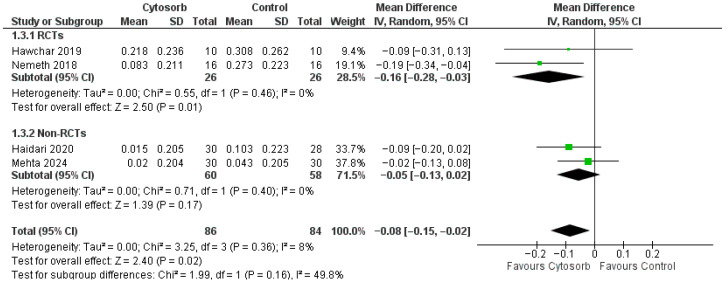
Forest plot for noradrenaline dosage. IV: inverse variance; CI: confidence interval [16,17,19,26].

As shown in Figure 2, Cytosorb treatment was significantly associated with a reduction in noradrenaline dosage with a MD of −0.08 [−0.15, −0.02] (*p* = 0.02, no heterogeneity-I2 8%). The inspection of funnel plots did not suggest publication bias. Subgroup analysis according to the study type showed that data coming from RCTs demonstrated a significant reduction in noradrenaline dosage (MD −0.16 [−0.28, −0.03], *p* = 0.01. On the contrary, no significant reduction is observed when including only data coming from non-RCTs (MD −0.05 [−0.13, 0.02], *p* = 0.17).

### 3.2. Mortality at Longest Follow Up

**Figure 3:**
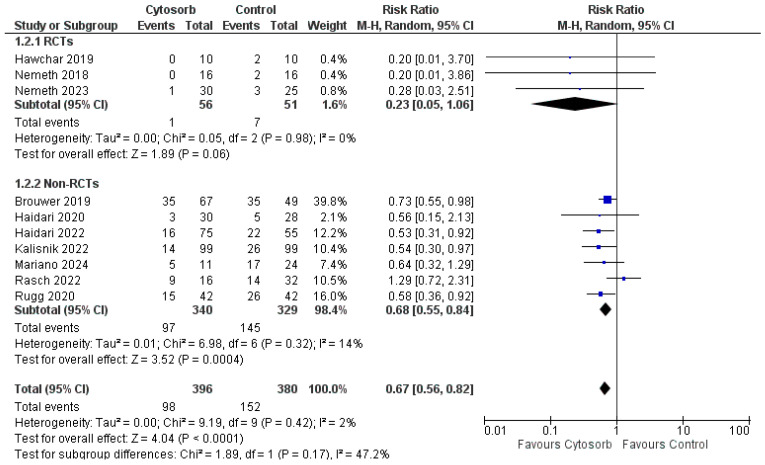
Forest plot for mortality at longest follow up. M-H: Mantel-Haenszel; CI: confidence interval [7,16,17,18,19,20,21,22,23,25].

Eleven studies reported data on mortality at the longest follow up. Among the studies reporting mortality outcomes, follow-up duration varied substantially. Brouwer et al. [7] assessed 28-day all-cause mortality as their primary endpoint. Haidari et al. [19,21] reported in-hospital mortality without specifying a fixed time point. Hawchar et al. [17] evaluated mortality at 24 h (primary outcome timepoint) with additional assessment at 48 h. Kalisnik et al. [22] conducted a follow-up through 30 days post-intervention. Mariano et al. [25] provided one of the longest follow-up periods at 270 days post-intervention. Nemeth et al. [16] assessed mortality at 30 days, while Nemeth et al. [23] extended follow-up to 1 year post-heart transplantation. Rasch et al. [20] evaluated 28-day mortality as well as ICU mortality and 1-year overall mortality. Rugg et al. [18] reported in-hospital mortality with a median length of stay of 25 days in the intervention group and 18 days in the control group. Patients receiving Cytosorb cartridge had a significantly lower mortality as compared to the control group (Figure 3), with a RR of 0.67 [0.56, 0.82] (*p* < 0.0001, no heterogeneity-I2 2%). No publication bias was detected by the analysis of the funnel plot. The subgroup analysis showed no significant difference between groups with data coming only from RCTs (RR 0.23 [0.05, 1.06], *p* = 0.06) and a significant improvement in survival with data coming from non-RCTS (RR 0.68 [0.55, 0.84], *p* = 0.0004).

### 3.3. ICU LOS

**Figure 4:**
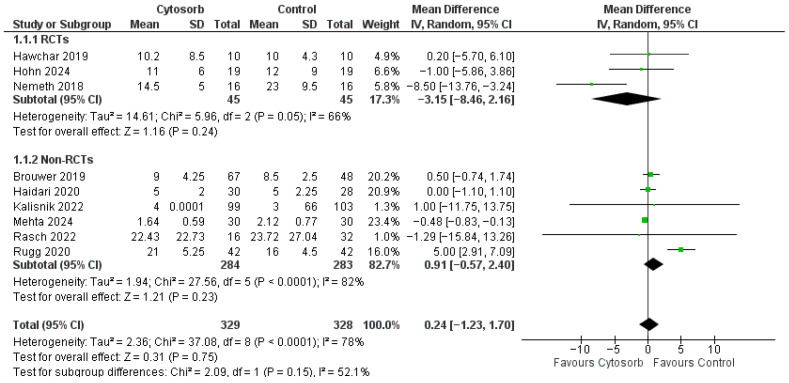
Forest plot for ICU LOS. IV: inverse variance; CI: confidence interval [7,16,17,18,19,20,22,24,26].

ICU LOS was reported in nine studies including critical adult patients (Figure 4). No differences were found between Cytosorb group and control group, with a MD of 0.24 [−1.23, 1.70] (*p* = 0.75, high heterogeneity-I2 78%). No publication bias was detected by the analysis of the funnel plot. Subgroup analysis according to the study design showed no differences between groups both with data coming from RCTs (MD −3.15 [−8.46, 2.16], *p* = 0.24) and from non-RCT studies (MD 0.91 [−0.57, 2.40], *p* = 0.23). The sensitivity analysis using the “leave-one-out at time” approach was performed on the primary outcome. The final result changed only when removing two studies from the analysis [16,23], with a *p* value ranging from 0.005 to 0.12.

**Figure 5:**
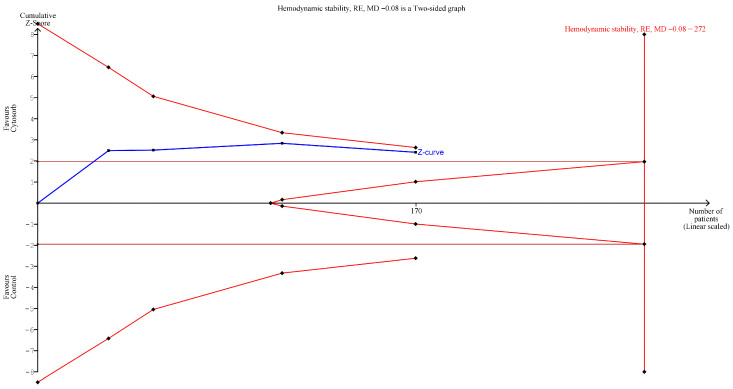
Trial sequential analysis for noradrenaline dosage outcome. RE: random effect model; MD: mean difference.

Risk of bias assessment was performed for all 12 of the included studies (Supplementary Material). For randomized controlled trials (n = 4), the Cochrane Risk of Bias 2 (RoB-2) tool was used, while the Newcastle–Ottawa scale (NOS) was applied for observational studies (n = 8). Among the RCTs, all studies demonstrated moderate overall risk of bias. The most common limitation was performance bias, as blinding of participants and personnel was not feasible due to the nature of the intervention (CytoSorb cartridge is visibly different from standard care). Nemeth et al. [16] and Hawchar et al. [17] showed moderate risk of selection bias due to unclear allocation concealment procedures, while Nemeth et al. [23] and Hohn et al. [24] implemented robust randomization methods, resulting in low selection bias. Detection bias was generally well-controlled in more recent trials, with Nemeth et al. [23] and Hohn et al. [24] employing blinded outcome assessors. Attrition and reporting biases were minimal across all RCTs, with complete follow-up data and pre-specified outcome reporting. For observational studies, the quality assessment using NOS revealed substantial methodological heterogeneity. Two studies [7,26] achieved NOS scores ≥6, indicating low risk of bias. Five studies [18,19,20,21,25] were classified as intermediate-risk (NOS scores 4–5). Selection bias was a significant concern in most observational studies, particularly those using retrospective designs without adequate matching procedures. Performance bias was uniformly high across observational studies due to the non-blinded nature of the intervention. Detection bias varied but was generally moderate, as most studies employed objective outcome measures like vasopressor dosage and mortality which are less susceptible to assessment bias. Attrition bias was minimal across studies, with most reporting complete follow-up data for the primary outcomes. The studies employing matching techniques [7,19,20] attempted to mitigate selection bias, though limitations persisted due to potential unmeasured confounders. Propensity score matching in Brower’s study [7] offered the most robust approach to control for baseline differences between intervention and control groups. The overall evidence quality, assessed using the GRADE methodology, was classified as low to very low for all outcomes (Supplementary Material). This classification reflects the predominance of observational studies, significant clinical heterogeneity, methodological limitations across included studies, and inconsistency in results for some outcomes. The evidence was downgraded due to risk of bias, indirectness (varied patient populations), and imprecision (wide confidence intervals in several analyses). As shown in Figure 5, the TSA on the primary outcome showed that the result is not robust as the Z-curve stands in the area between conventional and the alpha-spending boundary, with a ratio of patients recruited/needed of 170/272. The TSAs on mortality and ICU LOS were not feasible as the estimated sample size was too high to be represented.

## 4. Discussion

Our meta-analysis showed that: (1) treatment with CytoSorb is associated with a significant reduction in noradrenaline requirement; (2) though without a specific fixed timepoint of follow-up, patients treated with CytoSorb cartridge had a significantly lower mortality compared to the control group; (3) no effect on ICU LOS was found; (4) the overall evidence quality was low to very low for all outcomes.

Our findings must be interpreted within the context of recent meta-analyses examining CytoSorb efficacy, which have yielded apparently divergent conclusions. Becker et al. [29] analyzed 34 studies across heterogeneous conditions (sepsis, CPB surgery, COVID-19, cardiac arrest) and found no overall mortality benefit (RR 1.07 [0.88, 1.31]). Their sepsis subgroup (which included patients across the severity spectrum, not exclusively septic shock) also showed no significant reduction (RR 0.98 [0.74, 1.31]) [29]. The authors concluded that evidence did not support CytoSorb use for mortality reduction.

In contrast, Steindl et al. (2025) focused specifically on septic shock patients (nine studies, 744 patients) and reported significant reductions in hospital mortality (OR 0.64 [0.42, 0.97], *p* = 0.036) and 28–30 day mortality (OR 0.33 [0.19, 0.57], *p* < 0.001), closely aligning with our current findings [30].

This apparent contradiction likely reflects the critical importance of patient selection. The null findings in heterogeneous populations versus consistent benefits in rigorously defined septic shock cohorts suggest that CytoSorb efficacy is condition-specific rather than universal. The pathophysiology of cytokine dysregulation in post-CPB inflammation differs fundamentally from infectious septic shock; pooling these diverse conditions likely obscures real effects in specific populations.

Our analysis contributes several novel elements beyond Steindl et al.: (1) expanded evidence base with 12 studies versus nine, including 568 versus 449 CytoSorb-treated patients; (2) systematic evaluation explicitly linking hemodynamic stabilization to mortality outcomes, strengthening biological plausibility; (3) independent corroboration of their findings, which substantially increases confidence in the signal; and (4) contemporary contextualization within the current sepsis management frameworks.

The convergence of two independent meta-analyses specifically in septic shock provides the strongest evidence to date for potential benefit in this population. However, both analyses are limited by predominantly observational data, heterogeneous treatment protocols, and moderate risk of bias.

Our results confirm that the clinical utility of hemadsorption techniques in critically ill patients remains debated, due to the paucity of robust randomized controlled trials (RCTs) demonstrating a mortality benefit [31,32]. The CYTOSORB trial, which evaluated cytokine adsorption in patients with septic shock and acute lung injury, failed to demonstrate a significant reduction in IL-6 levels or improvement in multiple organ dysfunction syndrome [33]. Similarly, the CytoSorb registry showed variable effects on inflammatory markers and clinical outcomes across different patient populations [34].

It is conceptually debatable to infer that an adjunctive therapy might impact survival as long as there is no pathophysiological connection to the primary disease; rather, we can reasonably suppose that it might improve some of the accompanying phenomena that make patients sicker and more challenging to manage until disease-modifying therapies become effective [35,36]. The traditional focus on mortality as the primary endpoint may obscure potential benefits in intermediate physiological parameters that could translate to improved clinical management, even if not directly affecting survival [37].

Recent pathophysiological insights have highlighted the complex interplay between hemodynamic instability, inflammatory cascades, and organ dysfunction [38]. Circulating factors such as damage-associated molecular patterns (DAMPs), pathogen-associated molecular patterns (PAMPs), and various cytokines not only serve as biomarkers but actively contribute to vascular dysfunction, myocardial depression, and microcirculatory alterations [39,40]. This understanding provides a theoretical foundation for hemadsorption to attenuate these deleterious processes.

The concept of “hemodynamic coherence” (the correlation between macro- and microcirculatory parameters) has gained prominence in the critical care literature [41]. Interventions that restore macrohemodynamic parameters (e.g., mean arterial pressure, cardiac output) without addressing microcirculatory dysfunction may fail to improve outcomes [42]. By removing inflammatory mediators that impair vascular responsiveness and endothelial function, hemadsorption could potentially restore this coherence, improving tissue perfusion even in the absence of changes in conventional hemodynamic parameters [43,44].

The most significant finding of the present study is the reduction in vasopressor requirement. Elevated vasopressor doses have been consistently associated with adverse outcomes in critically ill patients, and the concept of “decatecholaminization” has gained increasing acceptance in recent years. Accordingly, the observed decrease in vasopressor demand may be regarded as a favorable, albeit preliminary, outcome.

Our meta-analysis yielded results that appear to contrast with those reported by Heymann et al. [45], who conducted a meta-analysis restricted to randomized controlled trials evaluating CytoSorb therapy in critically ill patients with inflammatory conditions, reporting increased mortality at longest follow-up. However, critical methodological differences limit direct comparability and necessitate cautious interpretation. When restricted to RCTs alone, our data demonstrated only a non-significant trend toward mortality reduction (RR 0.23 [0.05, 1.06], *p* = 0.06), consistent with Heymann’s findings. In contrast, observational studies showed significant mortality reduction (RR 0.67 [0.55, 0.82], *p* < 0.0001). This marked discrepancy strongly suggests that the apparent mortality benefit is substantially confounded by selection bias, indication bias, and unmeasured confounding inherent to non-randomized designs. Furthermore, trial sequential analysis revealed that current evidence has achieved only 62.5% of the required information size (170 of 272 patients), indicating insufficient statistical power for definitive conclusions. The Z-curve positioning between conventional and alpha-spending boundaries demonstrates that the observed mortality signal, while statistically significant by conventional meta-analysis, lacks robustness. This statistical fragility, combined with the predominance of observational data and low GRADE evidence quality, fundamentally undermines confidence in the mortality findings. The lower mortality observed should therefore be interpreted as hypothesis-generating rather than conclusive evidence of clinical benefit. While our findings argue against clinically meaningful harm, they cannot be interpreted as demonstrating definitive mortality benefit until confirmed by adequately powered, multicenter randomized controlled trials. Theoretical concerns remain regarding the potential removal of anti-inflammatory cytokines and certain antimicrobial agents, given that adsorption relies primarily on molecular weight rather than biological specificity. In contrast to mortality findings, the reduction in vasopressor requirements demonstrated greater consistency across study designs and may represent a more reliable signal, though this also requires validation in adequately designed trials with pre-specified hemodynamic endpoints before clinical recommendations can be formulated. This meta-analysis exhibits several notable strengths, including a comprehensive search strategy across multiple databases, adherence to established methodological guidelines with protocol registration on PROSPERO, and inclusion of both randomized and observational studies to maximize available evidence in a field where high-quality RCTs remain scarce. The focus on hemodynamic stability as a primary outcome addresses a clinically meaningful endpoint directly relevant to critical care practice, while the implementation of sensitivity analyses and trial sequential analysis enhances the robustness of our findings.

Nevertheless, important limitations must be acknowledged. Foremost among these is the significant clinical heterogeneity across included studies, with markedly different patient populations, intervention protocols, and control conditions potentially confounding interpretation of pooled results. The preponderance of observational studies with moderate to low methodological quality raises concerns about selection bias and unmeasured confounding, while inconsistent definitions of hemodynamic parameters and varying assessment timepoints compromise the precision of our estimates. The substantial statistical heterogeneity observed for ICU length of stay (I^2^ 77%) and the variable follow-up periods for mortality assessment further undermine confidence in these specific findings. Ultimately, the GRADE assessment of low to very low overall evidence quality necessitates cautious interpretation of our results and underscores the need for additional well-designed, adequately powered randomized trials to definitively establish the clinical utility of CytoSorb therapy in critically ill patients.

## 5. Conclusions

This meta-analysis demonstrates that CytoSorb hemoadsorption in septic shock is associated with significant reductions in vasopressor requirements and mortality, supporting a pathophysiologically plausible therapeutic effect through cytokine removal and hemodynamic stabilization. However, the predominantly observational evidence base, substantial heterogeneity in treatment protocols, and low overall evidence quality preclude definitive conclusions regarding clinical efficacy. CytoSorb therapy should therefore be considered investigational rather the than standard of care, with its use best reserved for rigorous clinical trials employing standardized protocols, biomarker-guided patient selection, and comprehensive assessment of both hemodynamic and long-term outcomes.

## Figures and Tables

**Table 1:** Characteristics of the included studies.

Author, Year	Design	Population	Sample Size per Group	Setting	Intervention	Control	Primary Outcome
Nemeth et al., 2018 [16]	RCT	Cardiac surgery with CPB	16/16	Single-center, cardiac surgery	CytoSorb during CPB	CPB	Vasoplegic syndrome, vasopressor requirements
Brouwer et al., 2019 [7]	Retrospective propensity-matched	Septic shock	67/49	Multi-center, ICU	CytoSorb	No Cytosorb	28-day all-cause mortality
Hawchar et al., 2019 [17]	RCT	Septic shock	20/20	Single-center, ICU	CytoSorb	No Cytosorb	Norepinephrine requirement
Rugg et al., 2020 [18]	Retrospective observational	Cardiogenic shock with VA-ECMO	18/18	Single-center, cardiac ICU	CytoSorb + VA-ECMO	VA-ECMO alone	Vasopressor requirements, inflammatory markers
Haidari et al., 2020 [19]	Retrospective matched	Native mitral valve infective endocarditis	15/30	Single-center, cardiac surgery	CytoSorb during CPB	CPB	Hemodynamic stability, SOFA score
Rasch et al., 2022 [20]	Matched cohort	Severe acute pancreatitis	16/32	Single-center, ICU	CytoSorb	No Cytosorb	Vasopressor dependency index/cardiac index improvement ≥20%
Haidari et al., 2023 [21]	Retrospective observational	Type A aortic dissection	25/25	Single-center, cardiac surgery	CytoSorb during CPB	CPB	Vasopressor support, inflammatory markers
Kalisnik et al., 2022 [22]	Prospective observational	High-risk cardiac surgery	30/30	Single-center, cardiac surgery	CytoSorb during CPB	CPB	Inflammatory markers, hemodynamic parameters
Nemeth et al., 2024 [23]	RCT	Heart transplantation	30/25	Single-center, cardiac surgery	CytoSorb during CPB	CPB	Vasoactive-inotropic score
Hohn et al., 2024 [24]	RCT	Elective cardiac surgery with CPB	19/19	Single-center, cardiac surgery	CytoSorb during CPB	CPB	IL-6 levels on ICU admission
Mariano et al., 2024 [25]	Retrospective observational	Burns with septic shock and AKI	11/24	Single-center, burn ICU	CytoSorb + CRRT	CRRT alone	Hemodynamic stability, survival
Mehta et al., 2024 [26]	Prospective observational	Elective aortic surgery	30/30	Single-center, cardiac surgery	CytoSorb during CPB	CPB	Vasoactive-inotropic score, SOFA score

CPB: ardiopulmonary bypass; RCT: Randomized Controlled Trial; ICU: Intensive Care Unit; VA-ECMO: Veno-arterial Extracorporeal Membrane Oxygenation; SOFA: Sequential Organ Failure Assessment; CRRT: Continuous Renal Replacement Therapy.

## Data Availability

No new data were provided.

## Supplementary Materials

The following supporting information can be downloaded at: https://www.mdpi.com/article/10.3390/life16040576/s1, Table S1: PRISMA Checklist; Table S2: PICOS Criteria; Table S3: Risk of Bias assessment; Table S4: GRADE of Evidence. Reference [46] is cited in the Supplementary Materials.
